# Effect of Phosalone on Testicular Tissue and *In Vitro* Fertilizing Potential 

**DOI:** 10.22074/ijfs.2015.4213

**Published:** 2015-04-21

**Authors:** Amir Amniattalab, Mazdak Razi

**Affiliations:** 1 Department of Pathology, Faculty of Veterinary Medicine, Urmia Branch, Islamic Azad University, Urmia, Iran; 2 Department of Comparative Histology and Embryology, Faculty of Veterinary Medicine, Urmia University, Urmia, Iran

**Keywords:** Phosalone, DNA Fragmentation, *In Vitro* Fertilization, Sperm, Testicular Tissue

## Abstract

**Background:**

The current study aimed to evaluate the effects of phosalone (PLN) as an organophosphate (OP) compound on testicular tissue, hormonal alterations and embryo development in rats.

**Materials and Methods:**

In this experimental study, we divided 18 mature Wistar rats into three groups-control, control-sham and test (n=6 per group). Animals in the test group received one-fourth the lethal dose (LD50) of PLN (150 mg/kg), orally, once per day for 45 days. DNA laddering and epi-fluorescent analyses were performed to evaluate testicular DNA fragmentation and RNA damage, respectively. Serum levels of testosterone and inhibin-B (IN-B) were evaluated. Testicular levels of total antioxidant capacity (TAC), total thiol molecules (TTM) and glutathione peroxidase (GSH-px) were analyzed. Finally, we estimated sperm parameters and effect of PLN on embryo development. Two-way ANOVA was used for statistical analyses.

**Results:**

There was severe DNA fragmentation and RNA damage in testicular tissue of animals that received PLN. PLN remarkably (p<0.05) decreased testicular TAC, TTM and GSH-px levels. Animals that received PLN exhibited significantly (p<0.05) decreased serum levels of testosterone and IN-B. Reduced sperm count, viability, motility, chromatin condensation and elevated sperm DNA damage were observed in the test group rats. PLN resulted in significant (p<0.05) reduction of *in vitro* fertilizing (IVF) potential and elevated embryonic degeneration.

**Conclusion:**

PLN reduced fertilization potential and embryo development were attributed to a cascade of impacts on the testicles and sperm. PLN promoted its impact by elevating DNA and RNA damages via down-regulation of testicular endocrine activity and antioxidant status.

## Introduction

Phosalone (PLN) or O-diethyl-s-(6-chloro-1-3 ben 30XO3ol-2(3H)-O-methyl) phosphorodithiote is known as an organophosphate (OP) compound used as a replacement for dichlorodiphenyltrichloroethane (DDT) in the agriculture and domestic animal fields (1). OP compounds exert their toxic impacts by blocking acetyl cholinesterase (an enzyme which dissipates acetyl choline) activities, which in turn result in accumulation of excessive amounts of acetyl choline in nervous tissue (2-4). Although these compounds have been known for their effect on the nervous tissue, several reports indicate that OP combinations adversely impact the genital system in both males and females. Lastly we have shown that glyphosate (an OP agent) reduced sperm quality and promoted energy dependent degeneration in germinal cells (5). Previous reports showed that the OP agents diazinon and malathion down-regulated expression of gene coding proteins involved in protein transcription (6, 7). Fattahi et al. (8) reported that administration of diazinon resulted in remarkable decreases in gonadotropin synthesis in rats and this mechanism significantly down-regulated testicular endocrine activity.

It has been shown that OP compounds partially exert their pathological impacts via promotion of oxidative stress in reproductive tissue (9,10). Accordingly, OP agents increase oxidants by disrupting enzymatic and/or non-enzymatic antioxidant defenses as well as enhancing high energy consumption coupled with inhibition of oxidative phosphorylation (5, 9). In addition, oxidative stress may cause degenerative alterations in sperm cells due to the high levels of polyunsaturated fatty acids (PUFA) in their plasma membrane (11). Imbalanced generation of oxidants affects the integrity of the sperm’s DNA by causing elevated frequencies of single strand DNA (SS-DNA) and double-strand DNA (DS-DNA) breaks (12).

However, the effect of PLN on testicular tissue, sperm parameters and *in vitro* fertilization (IVF) potential are unknown. Thus, we have aimed to estimate the effect of PLN on RNA and DNA damages in testicular tissue. The semen quality, sperm DNA fragmentation and IVF potential of sperm were analyzed. We also sought to analyze the testicular level of glutathione peroxidase (GSH-px), total antioxidant capacity (TAC) and total thiol molecules (TTM) in order to clarify any pathological alterations in testicular antioxidant capacity as well as illustrate the relationships of these factors with testicular degeneration and semen quality following PLN administration.

## Materials and Methods

### Chemicals

We obtained technical grade (9.8% purity) PLN [O, O- diethyl-s-(6-chloro-1-3 ben 30XO3ol- 2(3H)-O-methyl) phosphorodithiote] from Azma Chemical Ltd. (Tehran, Iran). Acridine-orange staining powder (Merck, Germany) and 3% hydrogen peroxide were purchased from Elim Teb Laboratory Kits Co., Ltd. (Urmia, Iran). The Ransol Detection Kit (Rondax Lab., Crumlin, BT 29, UK) for the GSH-px assay was also purchased from Elim Teb Laboratory Kits Co., Ltd. (Urmia, Iran).

### Animals and experimental design

To follow-up the current experimental study, 18 Wistar rats (200-220 g) were obtained from the animal resource of the Faculty of Veterinary Medicine, Urmia University. The animals were acclimatized for one week and had free access to food and water. The experimental protocols were approved by the Ethical Committee of Islamic Azad University, Urmia Branch in accordance with the Principles of Laboratory Animal Care.

The animals were randomly divided into three groups: control, control-sham and test. The animals in the control group received no chemical, whereas animals in the control-sham group received corn oil (1 cc daily by oral gavages). Lethal dose (LD50) values were determined by the Probit method (13). Animals in the test group received one fourth the LD50 of PLN (150 mg/kg dissolved in 1 cc corn oil) by oral gavages daily for 45 days (14).

### Histological analyses

After 45 days the animals were weighed and euthanized by a special CO_2_ device (Uromadaco, Iran). The testicular tissues were dissected free from surrounding tissues under high magnification (×40) stereo zoom microscope (model TL2, Olympus Co., Tokyo, Japan) and their weight was recorded. Dissected testes samples were washed with chilled normal saline and half of the specimens were fixed in Bouin’s fixative and kept for further histological analyses. The remaining samples were immediately frozen and stored at -70˚C for further biochemical analyses. Sections (5-6 μm) were stained with iron-Weigert Hematoxylin (Pajohesh Asia, Iran) for detection of germinal cell nuclei in the testis. The histological slides were analyzed under light microscope at two magnifications (×400 and ×1000). The tubular differentiation (TDI), repopulation (RI) and spermiogenesis (SPI) indices were evaluated from 20 sections of each sample. The results for percentage of tubules with positive TDI, RI and SPI were reported.

### Fluorescent analyses for RNA damage

RNA damage was assessed based on the Darzynkiewicz method (15). In brief, the testes were washed out with ether alcohol and cut by a cryostat (8 μm). The prepared sections were fixed by different degrees of alcohol for 15 minutes. Then, sections were briefly rinsed in 1% aqueous acetic acid followed by washing in distilled water. The specimens were subsequently stained in acridineorange (Sigma Aldrich, Germany) for 3 minutes and re-stained in phosphate buffer, followed by fluorescent color differentiations in calcium chloride. The degenerated cells were characterized by loss of RNA and/or by a faint red stained RNA. The normal cells were marked with bright red RNA close to the nucleolus. In order to reduce the bias problems, 20 sections for each sample were analyzed. We used an epi-fluorescent microscope (Model GS7, Nikon co., Japan) for imaging and analysis of the slides.

### Assessment of serum levels of testosterone and inhibin-B (IN-B)

After 45 days the blood samples were collected directly from the heart and allowed to clot at room temperature for 1 hour. Samples were centrifuged at 3000×g for 10 minutes to obtain the serum. The serum samples were stored at -80˚C for subsequent assays. Testosterone was assessed by a competitive chemiluminescent immunoassay kit (DRG Co, Germany). The serum level of inhibin-B (INB) was evaluated by an enzyme immunometric assay using a commercial kit (Pishtaz Teb, Iran).

### DNA laddering test

In order to examine for the presence of any DNA damage, we performed the qualitative DNA fragmentation assay on the frozen testis samples as previously described (16). Briefly, 0.2-0.3 g of frozen testis samples (pooled from at least 4 rats) from each individual group was homogenized in 3 ml lysis buffer (0.1 M tris-HCl/10 mM EDTA that contained 0.5% Triton X-100, pH=8.0). Following a short centrifugation (1200×g, 5 minutes at 4˚C), the pellets were treated with a mixture that contained buffer-saturated phenol, chloroform and isoamyl alcohol (25:24:1, v/v/v). After centrifugation (1500×g, 10 minutes at 4˚C), the supernatants were treated with a chloroform-isoamyl alcohol mix (49:1, v/v) to remove protein and fatty materials. Thereafter, to precipitate DNA, the solution was mixed with pre-chilled ethanol (absolute) and sodium acetate (3.5 M, pH=4.0), respectively. DNA samples were washed with ethanol (66%) and re-dissolved in buffer that contained tris-HCl (0.1 M) and EDTA (20 mM). DNA fragmentation was analyzed by loading the extracted DNA samples onto an agarose gel (1.6%) that contained ethidium bromide and electrophoresis was conducted at 60 V for 75 minutes. DNA fragmentation was imaged using a Gel Doc 2000 system (Bio-Rad).

### Evaluating epididymal sperm characteristics

The epididymis was carefully separated from the testicles under a ×20 magnification under a stereo zoom microscope (model TL2, Olympus Co., Tokyo, Japan). The epididymis was divided into three segments: caput, corpus and cauda. The epididymal cauda was trimmed and minced in 5 mL Ham’s F10 medium. After 20 minutes the minced epididymal tissue was separated from the released spermatozoa. The sperm count was performed according to standard hemocytometric lam method as described previously by Pant and Srivastava (17). We performed eosin-nigrosin staining to evaluate sperm viability. The sperm with stained head pieces were considered nonviable. Anilineblue staining was performed in order to analyze the sperm chromatin condensation. For this purpose, we prepared 20 smeared slides from each sperm sample of animals from different groups. The percentage of dead sperm was compared between different groups.

### Evaluating sperm motility

In order to evaluate sperm motility, the World Health Organization (1999) standard method for manual examination of sperm motility was used (18). Briefly, sperm samples were diluted (1:8) in Ham’s F10 prior to examination. A total of 20 μl of the sperm sample was placed on the sperm examination area and examined by a ×10 magnification loop. Only the motile sperm with forward progression was counted within ten boxes and recorded. Finally, motility was evaluated based on the following equation:

Motility (%)= [motile sperm/motile+non-motile sperm]×100

### Evaluating sperm DNA damage

To evaluate DNA double-strand breaks, air dried slides were stained with an acridine-orange staining kit (Sigma Co., St. Louis, MO, USA) after which the cover-slip was placed on the slides. The slides were evaluated on the same day using an epi-fluorescent microscope (model GS7, Nikon co., Japan). In all preparations at least 100 spermatozoa were evaluated at ×40 magnification. Spermatozoa with green fluorescence were considered to have native DS-DNA, the spermatozoa with yellow fluorescence were marked as having partly denatured SS-DNA (PSS-DNA), and with red fluorescence as completely denatured SS-DNA. Percentages of green, yellow, and red spermatozoa were assessed and compared between the groups.

### Sperm processing for *in vitro* fertilization

Samples that contained spermatozoa were prepared from the sperm suspensions as mentioned earlier. The samples were incubated at 37˚C under 5% CO_2_ (CO_2_ Incubator, LEEC, England) for 3 hours. Then, as previously described, 0.1 ml from superficial sperm and/or 0.1 ml from sediment sperm of suspensions in one tube were added to 150 μl of tissue culture medium (TCM) that contained the oocytes (19).

### Collection of oocytes and insemination

Eight mature female rats were injected subcutaneously with 7.5 IU pregnant mare’s serum (Netherlands) 48 hours prior to an intra-peritoneal injection of 100 IU human chorionic gonadotropin (hCG, Teikoku Zohki Co., Korea). Rats were euthanized with a special CO_2_ device 24 hours after the hCG injection. The oviducts were removed and the ampullar portion was placed into a plastic dish that contained phosphate buffered saline (pH=7.2). The oocytes in the cumulus masses were dissected out of the oviducts and introduced into TCM 199 (Sigma Co., USA). A drop of medium with 2 oocytes was allocated with a 10 μl sperm suspension (total: 80000 sperm) and incubated at 37˚C in 5% CO_2_ .

### Assessment of fertilization ratio and embryonic development

For this purpose, the appearance of pronuclei and polar bodies were checked under ×200 magnification using an inverted microscope (model NA100, Nikon CO., Japan). After 24 hours the two-cell embryo rate was assessed. *In vitro* embryonic development was evaluated at 120 hours by phase-contrast microscopy (model IX50, Olympus CO., Germany). Intact, fragmented and/or lysed embryos which did not develop were recorded as "arrested embryos". In the present study, the rate of cell lyses was recorded as follows: type I: fully lysed, necrotic and/or fragmented embryos, type II: embryos with partially lysed/fragmented blastomeres and type III: embryos with some lysed/ fragmented blastomeres and/or cytoplasmic vesicles (20).

### Assessment of serum total antioxidant capacity (TAC)

To determine the effect of PLN on oxidative stress, TAC of the testicular tissue from the controlsham and test groups were measured. The assessment was performed based on the ferric reduction antioxidant power (FRAP) assay (21). Briefly, at low pH (acetate buffer, 300 mM, pH=3.6), reduction of the FeIII-TPTZ complex to the ferrous form would produce an intensive blue color measurable at 593 nm. The intensity of the complex following addition of the appropriate volume of serum to the reducible solution of FeIII-TPTZ is directly related to the total reducing power of the electron donating antioxidant. An aqueous solution of FeII (FeSO4.7H2O) and appropriate concentration of freshly prepared ascorbic acid are used as blank and standard solutions, respectively.

### Measurement of serum total thiol molecules (TTM)

The total sulfhydryl level in testicular tissue was measured according to a method by Hu and Dillared (22). Briefly, 0.3-0.4 g of the testes samples were homogenized in ice-cold KCl (150 mM) after which the mixture was centrifuged at 3000×g for 10 minutes. Thereafter 0.5 ml of the supernatant was added to 0.6 ml tris-EDTA buffer (tris base 0.25 M, EDTA 20 mM, pH=8.2) followed by the addition of 40 μl DTNB (10 mM in pure methanol) in a 10 ml glass test tube. The final volume of the mentioned mixture was made up to 4.0 ml by extra addition of methanol. After incubation for 15 minutes at room temperature, the samples were centrifuged at 3000×g for 10 minutes and ultimately the absorbance of the supernatant was assessed at 412 nm.

### Assessment of glutathione peroxidase (GSH-px)

For this purpose, the testicular tissue was washed three times with 0.9% NaCl solution and 1.15% KCl was liquefied to the amount of 9 ml for each tissue. The homogenate of the tissues was prepared with a Teflon end on homogenizer (Elvenjem Potter, Newton, CT) and centrifuged at 4000 rpm for 5 minutes. The GSH-px activity was evaluated using a commercial Ransol measurement kit (Rondaxlab., Crumlin, BT 29, UK).

### Statistical analysis

Statistical analyses were performed using SPSS software version 13.00. The comparisons between groups were made by analysis of variance (twoway ANOVA) followed by the Bonferroni posthoc test. A p value <0.05 was considered significant. All values were expressed as mean±SD.

## Results

### Phosalone (PLN) reduced the total body and testicular weights

**Fig.1:**
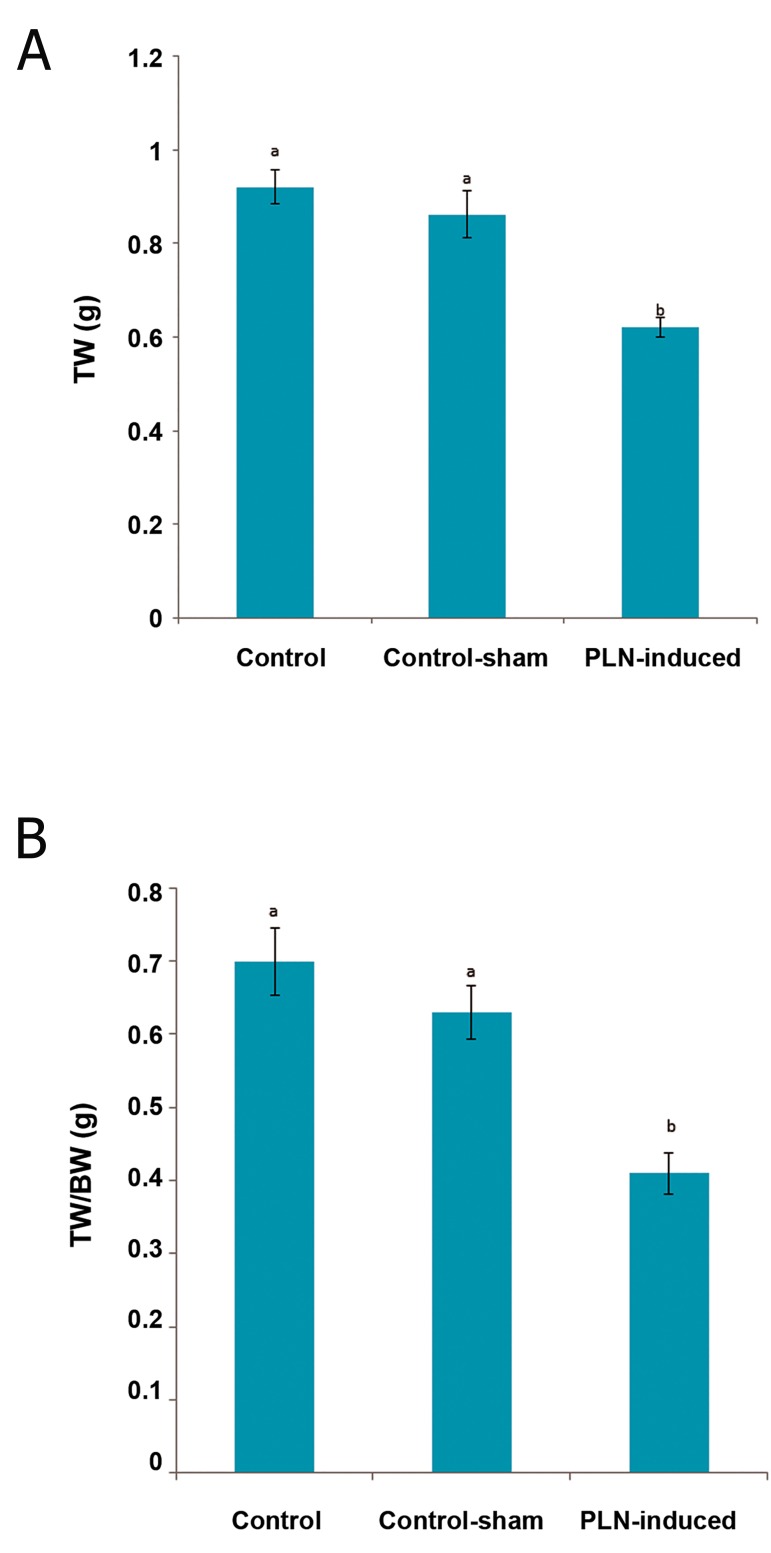
Effect of phosalone (PLN, 150 mg/kg) on (A) total testicular weight (TW) and on (B) testicular to body weight (BW) compared to control and control-sham groups. Data are mean±SD. ^a^; No significant and ^b^; Significant differences (p<0.05) between data for PLN-administered group with control and control-sham.

At the end of the study, observations demonstrated that the administration of PLN significantly (p<0.05) reduced the total body and testicular weight gains compared to control and controlsham groups. The testicular to body weight ratio in PLN-administered animals showed a remarkable (p<0.05) decrease compared to control and control-sham groups. No significant differences (p>0.05) were observed for total body and testicular weight gains between control and control-sham animals (Fig.1A-B).

### Phosalone (PLN) administration resulted in histological damages in testicular tissue

**Fig.2:**
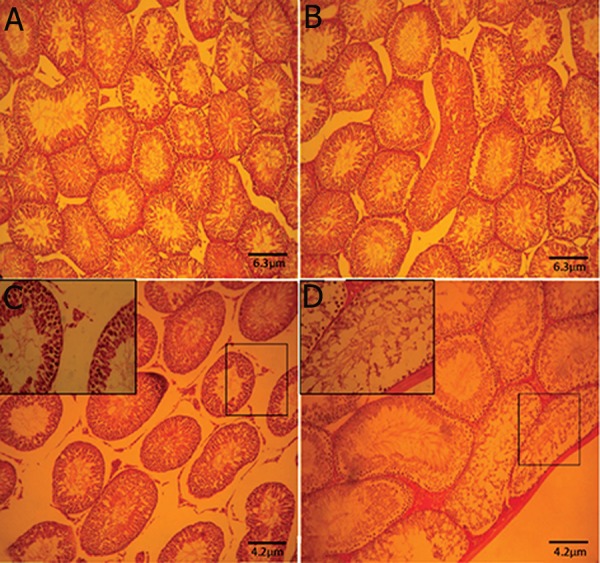
Cross-section from testis. (A) Control group. (B) Control-sham group: Note the normal seminiferous tubules with normal spermatogenesis. (C) and (D) Phosalone (PLN)-induced group: severe edema in connective tissue associated with tubular atrophy. Note the seminiferous tubules with negative tubular differentiation index (TDI) (magnified in figure C) and tubular depletion (TD) close to the capsule (magnified in figure D). Haematoxylin and eosin (H&E) staining (×400).

Histological analyses showed that the PLNadministered animals exhibited highly degenerated testicular tissue, an elevated percentage of tubules with arrested spermatogenesis (10.32 ±4.31%), severe edema in connective tissue and atrophied seminiferous tubules. Distribution of the Leydig cells remarkably (p<0.05) decreased and the percentage of hypertrophied Leydig cells increased per one mm^2^ of the interstitial tissue (Fig.2A-C). The PLN-treated animals showed an increased percentage of tubules with negative TDI, RI and SPI versus control and control-sham groups. No histopathological alterations were observed in control-sham animals. The data for histomorphometric analyses are presented in table 1.

**Table 1:** Histomorphometric alterations in different groups


	Control	Control-sham	PLN-induced

**T.D (µm)**	234.48±20.36^a^	228.52±19.07^a^	188.21± 18.88^b^
**G.E.H (µm)**	136.17±20.15^a^	137.8± 15.62^a^	106.07± 9.21^b^
**Negative TDI (%)**	14.20±2.07^a^	16.18± 1.66^a^	34.50±4.52^b^
**Negative RI (%)**	11.09±1.04^a^	13.17± 0.98^a^	28.74±6.01^b^
**Negative SPI (%)**	9.58±2.00^a^	8.33±1.26^a^	37.94±3.03^b^


PLN; Phosalone (150 mg/kg), T.D; Tubular diameter, G.E.H; Germinal epithelium height, TDI; Tubular differentiation index, RI; Repopulation index, SPI; Spermiogenesis index and ^a, b^; Significant differences (p<0.05) between PLN-induced group with control and control-sham groups (n=6 for each group). Data are mean±SD.

### Phosalone (PLN) significantly decreased serum levels of testosterone and inhibin-B (IN-B)

**Fig.3:**
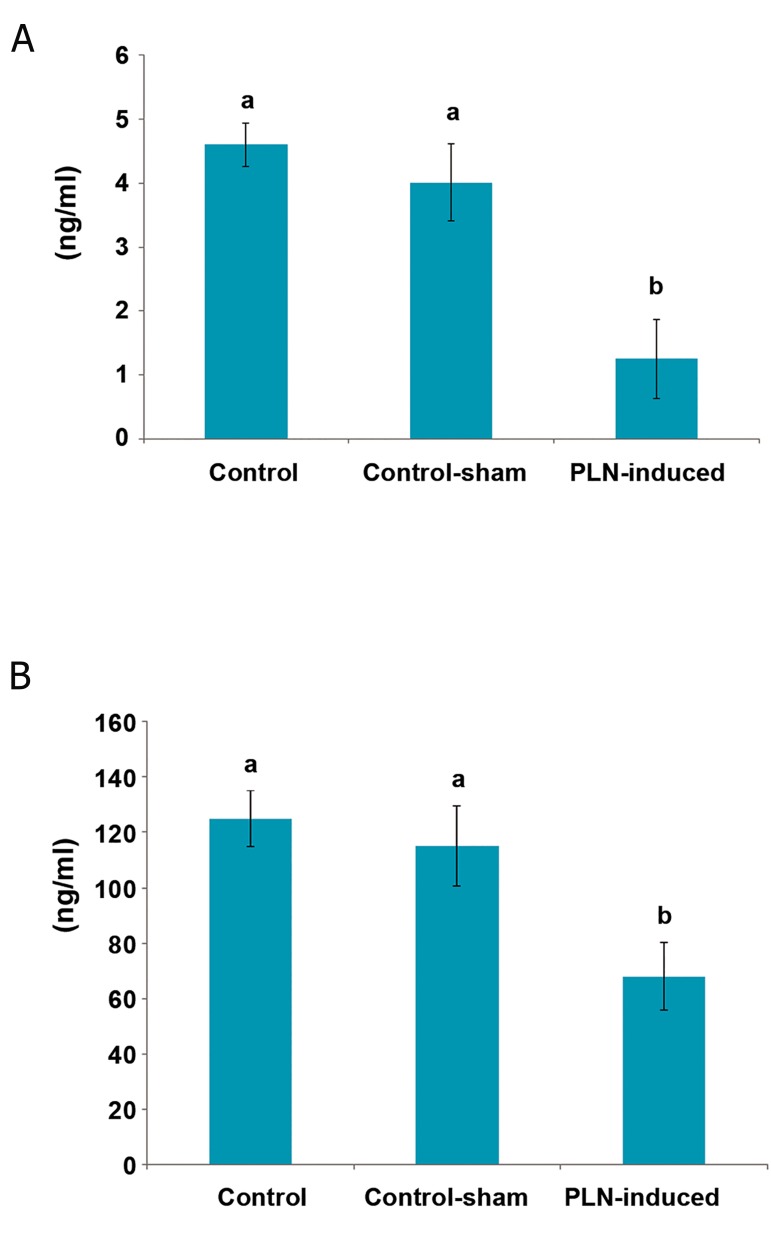
Effect of phosalone (PLN, 150 mg/kg) on serum levels of testosterone (A) and inhibin-B (IN-B) (B) compared to control and control-sham groups (n=6 rats for each group). Data are mean ±SD. ^a^; No significant and ^b^; Significant differences (p<0.05) between data for PLN-administered group with control and control-sham.

The serum levels of testosterone and IN-B significantly (p<0.05) reduced in PLN-treated animals versus control and control-sham groups. No significant alterations were observed in serum levels of testosterone and IN-B in the control- sham group compared to control animals (Fig.3).

### Exposure to phosalone (PLN) elevated the DNA and RNA damage in testicular tissue

**Fig.4:**
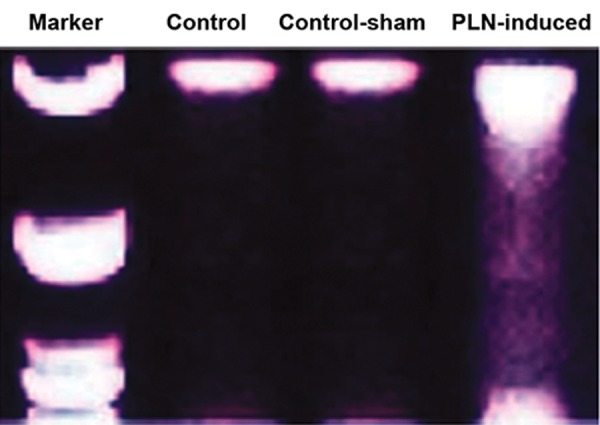
Phosalone (PLN)-induced DNA fragmentation in testicular tissue. PLN-induced DNA damage is shown as a smear shape in lane 4. No clear DNA fragmentation was observed in control and control-sham testicles, (DNA fragmentation assay).

**Fig.5:**
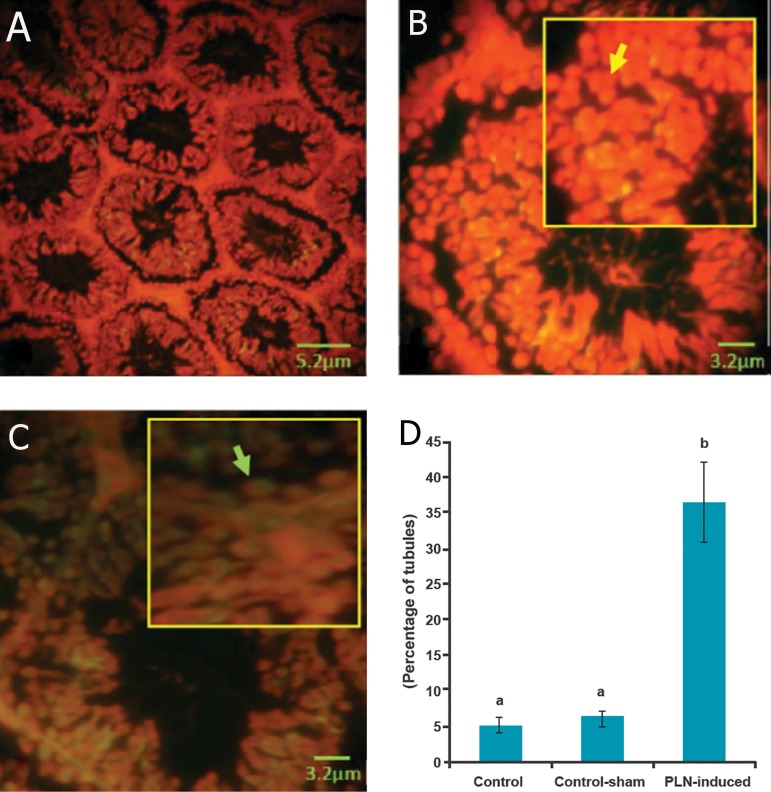
Cross-section from testis. (A) Control group. (B) Higher magnification from normal seminiferous tubule. Note the normal RNA content marked with bright, intensive fluorescent red in the germinal epithelium (yellow arrow). (C) Phosalone (PLN)-induced testis: remarkable RNA damage was shown by the faint fluorescent reaction (green arrow). The germinal cells exhibited lower RNA content versus normal cells in figure B. Epi-fluorescent analysis for RNA damage (A: ×400 and B, C: ×600). (D) Mean percentage of seminiferous tubules with RNA damage in different groups (n=6 rats per group). Data are mean±SD. ^a^; No significant and ^b^; Significant differences (p<0.05) between data for PLN-administered (150 mg/kg) group with control and controlsham groups.

Agarose gel electrophoresis was performed to examine for apoptotic DNA laddering. An accumulative dose of PLN after 45 days resulted in DNA degradation as characterized by a smear shape (Fig.4). Comparing the bands in the control group (lane 2, two relatively high molecular weight bands) with those in the PLN-treated group indicated that in the animals which received PLN, there was no proper high molecular weight DNA (lane 4). Epi-fluorescent analyses for RNA damage showed that chronic administration of PLN resulted in severe RNA damage in spermatocytogenesis and spermatogenesis cell lineages (Fig.5A-C). Accordingly, the animals that received PLN showed a significantly (p<0.05) higher percentage of seminiferous tubules with damaged RNA content in germinal cells (Fig.5D).

### Phosalone (PLN) reduced sperm quality

**Fig.6:**
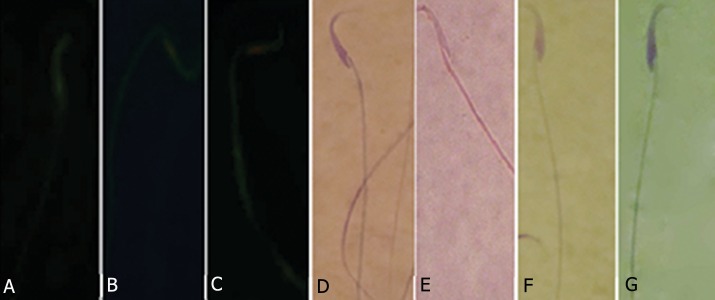
A. Sperm with double-strand DNA (DS-DNA). B. Sperm with partly denatured single-strand DNA (PSS-DNA). C. Sperm with singlestrand DNA (SS-DNA). D. Nonviable sperm with stained cytoplasm. E. Live sperm with colorless cytoplasm. F. Sperm with condensed chromatin. G. Sperm with immature chromatin condensation. A-C. Acridine-orange stain, D, E. Eosin-nigrosin stain and F, G. Aniline-blue stain.

The PLN-administered animals showed a significant (p<0.05) decrease in sperm count (48.25±6.21×10^6^) compared to control-sham (63.51±3.70×10^6^) and control (66.12±4.41×10^6^) groups. The animals in the PLN-treated group exhibited a significantly (p<0.05) higher percentage of dead sperms versus the control- sham and control animals. The percentage of sperms with PSS-DNA and SS-DNA were remarkably (p<0.05) elevated in PLN-administered animals in comparison to control and control-sham animals. Moreover, the PLN-administered animals showed a significant (p<0.05) reduction in the percentage of sperms with condensed chromatin (Fig.6). The data for sperm parameters are presented in table 2.

**Table 2:** Effect of phosalone (PLN) on sperm parameters in different groups


	Control	Control-sham	PLN-induced

**Count (×10^6^)**	66.12± 4.41^a^	63.51± 3.70^a^	48.25±6.21^b^
**Viability (%)**	83.45± 11.10^a^	80.00± 8.12^b^	40.15±6.45^b^
**Motility (%)**	85.12± 9.21^a^	82.35± 10.12^a^	34.41±6.52^b^
**Chromatin condensation (%)**	86.42± 6.74^a^	80.32± 4.63^a^	38.64±3.10^b^
**DS-DNA (%)**	82.32± 6.14^a^	81.11±7.33^a^	38.21±5.12^b^
**PSS-DNA (%)**	10.07± 1.01^a^	12.31± 2.10^a^	27.81±3.71^b^
**SS-DNA (%)**	8.33±0.78^a^	10.28± 0.84^a^	34.63±2.01^b^


PLN; Phosalone (150 mg/kg), DS-DNA; Double-strand DNA, PSS-DNA; Partial single-strand DNA, SS-DNA; Single-strand DNA and ^a, b^; Significant differences (p<0.05) between PLN-induced group with control and control-sham groups (n=6 for each group) in the same row. Data are mean±SD.

### Phosalone (PLN) reduced *in vitro* fertilizing (IVF) potential

**Fig.7:**
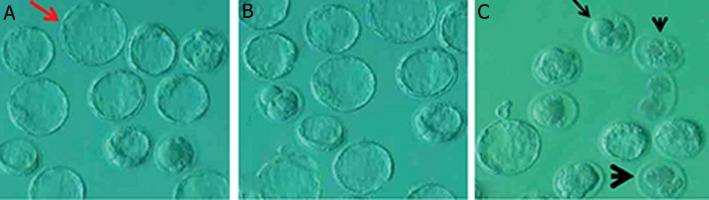
Embryonic development in (A) control, (B) control-sham and (C) phosalone (PLN)-induced animals. Control and control-sham groups exhibited significantly higher normal blastocysts (red arrow). The PLN-induced animal exhibited remarkably higher type I (head arrow), type II (big head arrow) and type III arrests (arrow).

The results for IVF of oocytes by sperm collected from PLN-administered animals were remarkably (p<0.05) lower than control and control-sham animals. Interestingly, the significantly (p<0.05) higher percentage of 2-cell embryos stopped division in animals that received PLN and did not continue. Comparing the percentage of blastocysts between different groups showed that the PLN-treated animals exhibited a significantly (p<0.05) lower percentage of blastocyst versus the control and control-sham animals. No significant differences (p>0.05) were observed between control and control-sham animals (Fig.7). The data for IVF results are presented in table 3.

### Phosalone (PLN) reduced antioxidant status and elevated oxidative stress

**Fig.8:**
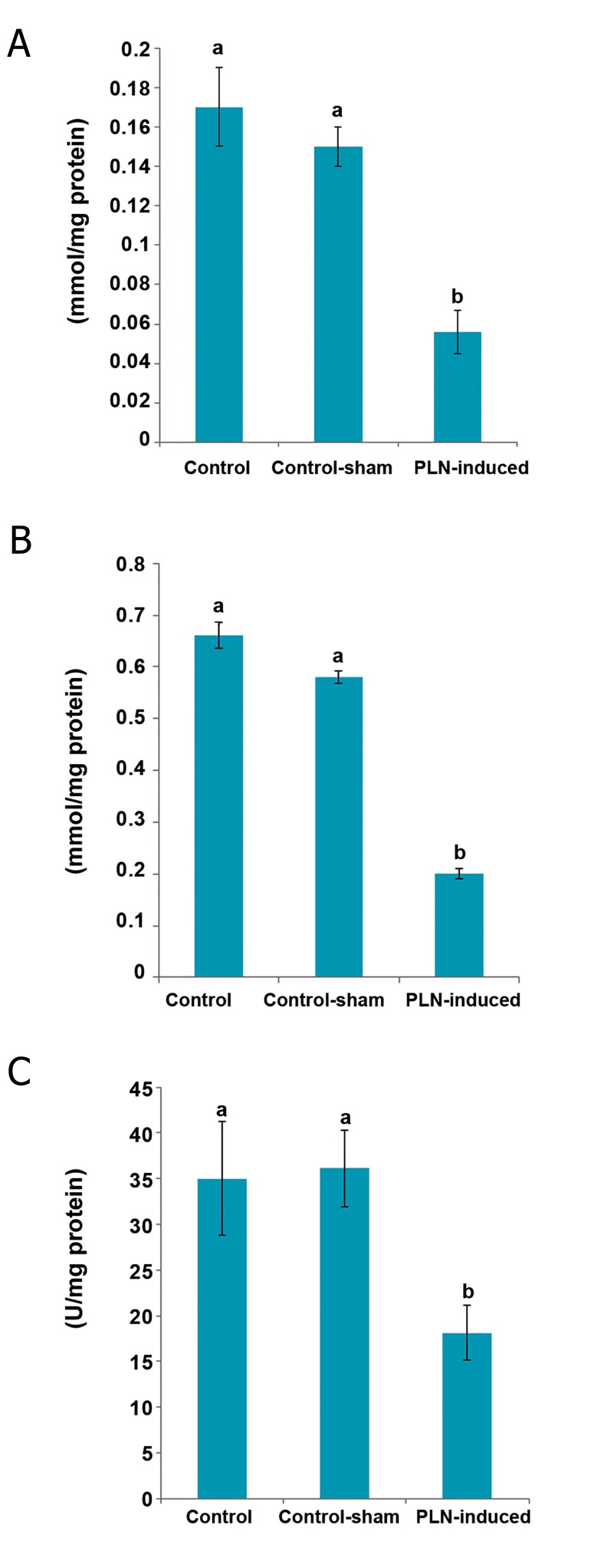
Effect of phosalone (PLN) on tissue (A) total antioxidant capacity (TAC), (B) total thiol molecules (TTM) and (C) glutathione peroxidase (GSH-px) compared to control and control-sham groups (n=6 rats for each group). Data are mean±SD. ^a^; No significant and ^b^; Significant differences (p<0.05) between data for PLN-administered group with control and control-sham groups.

Observations demonstrated that the tissue levels of TAC and TTM significantly down-regulated in the PLN-administered group compared to control and control-sham animals. Biochemical analyses for GSH-px revealed that the animals in the group that received PLN exhibited a remarkable (p<0.05) decrease in testicular GSH-px levels (Fig.8A-C).

**Table 3:** Effect of phosalone (PLN) on *in vitro* fertilizing (IVF) potential and embryo development


	Control	Control-sham	PLN-induced

**Total oocytes (NO)**	203	277	281
**Appropriate oocyte (NO)**	170	162	63
**Fertilized oocyte (NO)**	148	146	37
**2-cell embryos (%)**	81.03±7.14^a^	78.64±5.17^a^	64.30±6.85^a^
**Blastocysts (%)**	54.28±3.70^a^	47.21±4.01^a^	20.22±2.90^b^
**Arrested embryos (%)**	42.51±5.20^a^	48.63±4.21^a^	68.33±4.83^b^
**Arrest type I (%)**	4.21±0.98^a^	4.68±1.01^a^	27.35±4.43^b^
**Arrest type II (%)**	8.63±1.41^a^	7.36±1.33^a^	23.72±3.10^b^
**Arrest type III (%)**	31.21±2.84^a^	28.40±4.71^a^	18.70±2.69^b^


^a, b^; Significant differences (p<0.05) between PLN-induced group with control and control-sham groups (n=6 for each group) in the same row. Data are mean±SD.

## Discussion

The results of the present study showed that chronic administration of PLN resulted in severe damages to testicular tissue. The animals that received PLN had remarkable DNA fragmentation, RNA damage and down-regulated intra-testicular endocrine activities. The enzymatic and non-enzymatic antioxidant potentials were down-regulated after 45 days. Finally, the PLN-administered animals exhibited a remarkable reduction in IVF outcomes. PLN-treated animals showed significantly higher embryonic arrests versus control and control-sham animals. 

Previous reports indicated that chronic administration of different OP compounds (glyphosate, diazinon and malathion) resulted in severe reductions in gonad weights in rats (5,7). Severe testicular damages were considered to be a possible explanation for this impairment. Animals in the PLN-treated group had a significant reduction in total body weight gain. This alteration might be related to the effect of PLN on the central structures involved in the control of feed intake such as the ventromedian nucleus of the hypothalamus (23). 

Previous reports showed that OP combinations affected the spermatogenesis process and impacted the male reproductive system via interruption of endocrine activities (24,25). In physiologic conditions, the interactions between Leydig and Sertoli cells promote the spermatogenesis process (26). Accordingly, Leydig cells control the Sertoli cell endocrine activities via synthesis of testosterone (26,27). In this regard, PLN-treated animals have shown a significant reduction in Leydig cell numbers per one mm ^2^ of the interstitial tissue as well as decreased serum levels of testosterone and IN-B. We can suggest that PLN has influenced the testicular endocrine status by decreasing distribution of Leydig cells, which in turn disrupted the Leydig stimulatory impact on Sertoli cell endocrine interactions. The elevated testicular damages such as negative RI, TDI and SPI reflect the functional derangement of the Sertoli cells. The decreased testicular to body weight ratio in animals that received PLN could be attributed to severe degeneration of testicular tissue. 

Because of the high concentration of unsaturated fatty acids in mammalian germinal cells, these cells are susceptible to oxidative stress (28, 29). The produced oxidative stress results in considerable damages at lipids, proteins, DNA and RNA levels (12,30,31). In order to evaluate the effect of PLN on testicular antioxidant status, the testicular levels of TTM, GSH-px and TAC have been analyzed. Observations showed remarkable reductions in TTM, GSHpx and TAC levels in PLN-treated animals compared to control and control-sham groups. The intracellular antioxidant enzymes GSH-px and superoxide dismutase are known for the first line of cellular defense that prevent oxidative stress-induced damages (11,28). GSH-px plays an important role in maintenance of the thiol-disulfide balance. Any lack in GSH-px physiological interactions and/or reduction in GSH-px synthesis will affect the enzymatic defense line and ultimately promote damages on biological macromolecules such as DNA and RNA (11,28,32). Our analyses have shown elevated DNA fragmentation and RNA damage as well as decreased testicular GSH-px levels in PLN-administered animals. Increased apoptotic DNA fragmentation and severe RNA damage in PLN-treated animals enabled us to conclude that remarkable depletion in GSH-px associated with decreased TTM level could promote the oxidative stress-induced damages at DNA and RNA levels. 

It has been well established that sperm DNA integrity is very important for its fertilizing potential. The DNA integrity of sperm mainly depends on its compaction after chromatin condensation processes (11,33). The deficiency in protamine expression and/or replacement with nucleosomal histones caused by environmental toxicants promotes DNA disintegrity because decondensed DNA of the sperm are susceptible to free radicals (32). Our observations have demonstrated that the percentage of sperms with condensed chromatin decreased and there was increased DNA damage in animals treated with PLN. Therefore, we concluded that PLN appended the sperm DNA disintegrity both by affecting the chromatin condensation process and by down-regulating antioxidant status. More analyses showed that PLN administration resulted in diminished sperm motility and viability. The sperm cell membrane contains high amounts of PUFA which are susceptible to exogenous free radicals (24). Therefore, it is logical to suggest that reduced sperm viability reflects severe oxidative stress in PLN-administered animals. Beside reduced viability, the PLN-induced oxidative stress can inhibit axonemal protein phosphorylations (34,35) and consequently reduce sperm motility. 

The cascade of events PLN-increased percentage of dead sperms associated with elevated DNA damages, as well as immobility and nuclear immaturity are able to enhance oxidative stress. Accordingly, the damaged sperm are considered as the sources of radicals (36). It has been shown that incubation of abnormal and/or damaged spermatozoa lead to oxidative damages at the DNA level in oocytes and embryos (37). It is reported that oocytes have the ability to correct small scale DNA damage upon fertilization. If this increases above a certain level it may be difficult for the oocyte to cope and lead to fertilization failure or impaired embryo development (38). In corroboration with this finding we have observed that embryo development significantly decreased in PLN-treated animals. A possible explanation may be that the increased oxidants (produced from abnormal sperms) possibly led to remarkable DNA damage in oocytes, which in turn resulted in lower blastocyst generation. On the other hand, PLN resulted in an elevated percentage of sperm with abnormal chromatin condensation. Previous reports indicated that using sperm with decondensed DNA resulted in low *in vitro* embryo development, particularly at the 2-cell embryo level (38).Therefore, it could be suggested that decreased embryo development in PLN-administered animals could be attributed to increased damage at sperm levels such as reduced sperm motility, viability, chromatin condensation and increased DNA damage. 

## Conclusion

The results of the current study showed that chronic exposure to PLN resulted in enhanced DNA fragmentation and RNA damage in testicular tissue and reduced testicular endocrine activities. Our observations demonstrated that PLN exerted its impact via down-regulation of the antioxidant status and enhanced oxidative stress. Finally, we showed that PLN-induced problems in sperm parameters resulted in considerable embryo toxicity. 

## References

[1] Singh AK (1985). Kinetic analysis of inhibition of brain and red blood cell acetylcholinesterase and plasma cholinesterase by acephate or methamidophos. Toxicol Appl Pharmacol.

[2] Harlin KS, Dellinger JA (1993). Retina, brain and blood cholinesterase levels in cats treated with oral dichlorvos. Vet Hum Toxicol.

[3] Bustos-Obregon E, Gonzalez JR, Espinoza O (2005). Melatonin as protective agent for the cytotoxic effects of diazinon in the spermatogenesis in the earthworm Eisenia foetida. Ital J Anat Embryol.

[4] Pina-Guzman B, Solis-Heredia MJ, Quintanilla-Vega B (2005). Diazinon alters sperm chromatin structure in mice by phosphorylating nuclear protamines. Toxicol Appl Pharmacol.

[5] Razi M, Najafi G, Feyzi S, Karimi A, Shahmohamadloo S, Nejati V (2012). Histological and histochemical effect of Gly-Phosate on testicular tissue and function. Iran J Reprod Med.

[6] Bonilla E, Hernández F, Cortés L, Mendoza M, Mejía J, Carrillo E (2008). Effects of the insecticides malathion and diazinon on the early oogenesis in mice *in vitro*. Environ Toxicol.

[7] Adamkovicova M, Toman R, Cabaj M (2010). Diazinon and Cadmium acute testicular toxicity in rats examined by histopathological and morphological methods. Slovak J Anim Sci.

[8] Fattahi E, Parivar K, Jorsaraei SGA, Moghadamnia AA (2009). The effects of diazinon on testosterone, FSH and LH levels and testicular tissue in mice. Iran J Reprod Med.

[9] Harris C, Lee E, Hiranruengchok R, McNutt TL, Larson SJ, Akeila S (1996). Characteristics of glutathione redox and antioxidant status in post implantation rat embryos: response to oxidative stress. Toxicology.

[10] Milatovic D, Gupta RC, Aschner M (2006). Anticholinesterase toxicity and oxidative stress. ScientificWorldJournal.

[11] Agarwal A, Allamaneni S, Singh K (2006). Oxidative stress and human reproduction. Oxidative stress, disease and cancer.

[12] Fraga CG, Motchnik PA, Wyrobek AJ, Rempel DM, Ames BN (1996). Smoking and low antioxidant levels increase oxidative damage to sperm DNA. Mutat Res.

[13] Finney DJ (1971). Probit analysis.

[14] Zama D, Meraihi Z, Boubekri N, Amrani A, Tebibel S, Baali N (2005). Assesment of the changes in some diagnostic enzymes and other parameters in wistar albino rats treated with pesticides during gestation. Sci Technol.

[15] Darzynkiewicz Z (1990). Differential staining of DNA and RNA in intact cells and isolated cell nuclei with acridine orange. Methods Cell Biol.

[16] Patel N, Joseph C, Corcoran GB, Ray SD (2010). Silymarin modulates doxorubicin-induced oxidative stress Bcl-xL and p53 expression while preventing apoptotic and necrotic cell death in the liver. Toxicol Appl Pharmacol.

[17] Pant N, Srivastava SP (2003). Testicular and spermatotoxic effect of quinaphos in rats. J Appl Toxicol.

[18] World Health Organization (2000). WHO laboratory manual for the examination of human semen and sperm-cervical mucus interaction.

[19] Toyoda Y, Chang MC (1974). Fertilization of rat eggs *in vitro* by epididymal spermatozoa and the development of eggs following transfer. J Reprod Fertil.

[20] Cebral E, Carrasco I, Vantman D, Smith R (2007). Preimplantation embryotoxicity after mouse embryo exposition to reactive oxygen species. Biocell.

[21] Benzie IF, Strain JJ (1999). Ferric reducing/antioxidant power assay: direct measure of total antioxidant activity of biological fluids and modified version for simultaneous measurement of total antioxidant power and ascorbic acid concentration. Methods Enzymol.

[22] Hu M, Dillared CJ (1994). Plasma SH and GSH measurement. Method Enzymol.

[23] Psychoyos A, Meyer P (1983). La reproduction. Physiologie Humaine.

[24] Kamijima M, Hibi H, Gotoh M, Taki K, Saito I, Wang H (2004). A survey of semen indices in insecticide sprayers. J Occup Health.

[25] Krause W, Hamm K, Weissmulter J (1975). The effect of per orally administered DDVP and Malathion on spermatogenesis and Leydig cell in the Juvenile rat. Andrologia.

[26] Skinner MK, Fritz IB (1985). Testicular peritubular cells secrete a protein under androgen control that modulates Sertoli cell function. Proc Natl Acad Sci USA.

[27] Walker W, Cheng J (2005). FSH and testosterone signaling in Sertoli cells. Reproduction.

[28] Yamanaka K, Hasegawa A, Sawamura R, Okada S (1991). Cellular response to oxidative damage in lung induced by the administration of dimethylarsinic acid, a major metabolite of inorganic arsenics, in mice. Toxicol Appl Pharmacol.

[29] Vernet P, Aitken RJ, Drevet JR (2004). Antioxidant strategies in the epididymis. Mol Cell Endocrinol.

[30] Sega GA (1991). Adducts in sperm protamine and DNA vs.mutation frequency. Prog Clin Biol Res.

[31] Recio R, Robbins WA, Borja-Aburto V, Moran-Martınez J, Froines JR, Hernandez RM (2001). Organophosphorous pesticide exposure increases the frequency of sperm sex null aneuploidy. Environ Health Perspect.

[32] Usoh IF, Akpan EJ, Etim EO, Farombi EO (2005). Antioxidant actions of dried flower extracts of Hibiscus sabdariffa L.On sodium arsenite-induced oxidative stress in rats. PJN.

[33] Sakkas D, Urner F, Bizzaro D, Manicardi G, Bianchi PG, Shoukir Y (1998). Sperm nuclear DNA damage and altered chromatin structure: effect on fertilization and embryo development. Hum Reprod.

[34] Green DR, Reed JC (1998). Mitochondria and apoptosis. Science.

[35] Twigg J, Irvine DS, Houston P, Fulton N, Michael L, Aitken RJ (1998). Iatrogenic DNA damage induced in human spermatozoa during sperm preparation: protective significance of seminal plasma. Mol Hum Reprod.

[36] Said TM, Agarwal A, Sharma RK, Thomas AJ, Sikka SC (2005). Impact of sperm morphology on DNA damage caused by oxidative stress induced by beta-nicotinamide adenine dinucleotide phosphate. Fertil Steril.

[37] Nasr-Esfahani MH, Aitken JR, Johnson MH (1990). Hydrogen peroxide levels in mouse oocytes and early cleavage stage embryos developed in vitro or in vivo. Development.

[38] Sakkas D, Mariethoz E, Manicardi G, Bizzaro D, Bianchi PG, Bianchi U (1999). Origin of DNA damage in ejaculated human spermatozoa. Rev Reprod.

